# Maraviroc – A CCR5 Antagonist for the Treatment of HIV-1 Infection

**DOI:** 10.3389/fimmu.2015.00277

**Published:** 2015-06-05

**Authors:** Elna Van Der Ryst

**Affiliations:** ^1^The Research Network, Sandwich, UK

**Keywords:** HIV, CCR5 antagonist, HIV-1 RNA, tropism, antiretroviral drug

Despite the dramatic decline in human immunodeficiency virus type 1 (HIV-1)-related morbidity and mortality following the discovery of the protease inhibitors and the advent of combination highly active antiretroviral (ARV) therapy in the mid-1990s, many patients were still failing therapy due to resistance and/or intolerability (1). It was clear that more ARVs acting on different steps in the virus lifecycle, active against resistant viruses, and better tolerated were needed. The demonstration of the key role of the chemokine receptors CCR5 and CXCR4 in HIV-1 entry sparked interest in this process as a new ARV target (2, 3). CCR5 is the co-receptor for the majority of HIV-1 strains, and these viruses are termed CCR5 tropic (R5). Virus strains that use CXCR4 are called CXCR4-tropic (X4), while strains that can use both receptors are dual-tropic (4). Virus from a patient can often contain mixtures of R5, X4, and dual-tropic strains, collectively called CXCR4-using.

The key role of CCR5 in HIV-1 entry, coupled with the demonstration that individuals who were homozygous for a 32 base pair deletion in the CCR5 gene (CCR5Δ32), and subsequently do not express functional CCR5, were highly protected from infection with R5 HIV-1, focused attention on CCR5 as an attractive target (5). Although some studies have demonstrated subtle effects of the CCR5Δ32 mutation on immune function, such as decreased inflammatory scores in hepatitis C-infected individuals and recovery from hepatitis B in heterozygotes; while homozygotes are more susceptible to tick-borne encephalitis and severe West Nile virus disease, these individuals suffer little apparent adverse effects on their health (5, 6). This, together with the fact that members of the G protein-coupled receptor superfamily are often tractable to development of potent, selective, and orally bioavailable drugs (7), led to the initiation of CCR5 ligand discovery programs by multiple groups, including a team from Pfizer Global Research and Development based at the Sandwich laboratories in the United Kingdom.

Maraviroc (UK-427,857, MVC) was discovered through high-throughput screening of the Pfizer compound library using a chemokine radioligand-binding assay. The most promising compound from the screening process was optimized for potency against the receptor, antiviral activity, pharmacokinetic characteristics, and selectivity against human cellular targets through a large medicinal chemistry effort in which almost 1000 molecules were characterized (7). MVC binds in the transmembrane pocket of CCR5 and is a slow-offset functional antagonist that prevents internalization (7, 8). It has potent antiviral activity against a wide-range of HIV-1 isolates (7). Together with its excellent pre-clinical safety profile and acceptable pharmacokinetics, this resulted in it being nominated as a clinical candidate in December 2000 (7).

It was always clear that the clinical development of CCR5 antagonists would be challenging, as these would be the first host-targeted ARV drugs and we were therefore venturing into uncharted territory. In order to pre-empt key issues, a clinical development team was established very soon after the start of the discovery program and I was recruited to lead the early development team, joining Pfizer in February 1999. We identified several key challenges to address in the design of the clinical program, in addition to demonstrating safety and efficacy. The first of these was that no commercially available, clinically validated assay to identify patients infected with R5 HIV-1 existed. This was critical, as MVC is active only against R5 HIV-1 strains (7). Secondly, in spite of the apparently healthy phenotype of individuals with CCR5Δ32 (5, 6), concerns remained regarding the safety of long-term exposure to CCR5 antagonists, as blocking of CCR5 may be different from congenital absence of the CCR5 receptor, where the immune system has matured in the absence of CCR5 and compensatory mechanisms may have developed. Finally, in HIV-1 infected individuals, the incidence of CXCR4-using HIV-1 strains increases with disease progression and decrease in CD4 cell counts (9), although no causal link between CXCR4-using virus and CD4-cell depletion has been demonstrated. This has led to concerns that selective pressure from a CCR5 antagonist may drive the virus population to use CXCR4 and result in CD4 cell decline.

Phase 1 single and multiple dose studies in healthy volunteers, conducted in 2001 and the first half of 2002, demonstrated that MVC was safe and well-tolerated in multiple doses up to 300 mg twice a day (BID), had a pharmacokinetic profile compatible with once daily (QD) or BID oral dosing, could be combined with other ARVs, and that doses of ≥100 mg BID resulted in exposure above the geometric mean antiviral IC_90_ *in vitro* (7, 10). To demonstrate proof of pharmacology, CCR5 receptor saturation was measured using a bespoke *ex vivo* MIP-1β internalization assay. Dose-dependent saturation was demonstrated, with doses of ≥25 mg QD resulting in near maximum saturation levels, raising the interesting possibility that MVC could be efficacious in doses as low as 25 mg QD. Receptor saturation remained high for several days after dosing was discontinued, reflecting slow offset from the receptor *in vivo* (11).

We were both excited and encouraged by the phase 1 data and rapidly moved on to a phase 2a proof of concept program. HIV-1 infected patients were screened for the presence of R5 virus only, using a novel phenotypic tropism assay (Trofile^®^, Monogram Biosciences, South San Francisco, CA, USA), and received MVC as monotherapy for 10 days (12). CCR5 receptor saturation was measured in this study to evaluate the possibility of using this as a biomarker for efficacy and in therapeutic monitoring. The keenly awaited data lived up to our expectations and demonstrated that doses of ≥100 mg BID resulted in mean maximum HIV-1 RNA reductions of >1.5log_10_ (Figure 1A), with all patients, excluding one patient with X4 virus who has been erroneously included, achieving an HIV-1 RNA reduction of at least 1log_10_ (12). This gave us confidence that the assay correctly identified patients likely to respond to MVC. HIV-1 RNA nadir occurred 1–5 days after the last dose of MVC, consistent with prolonged receptor saturation as demonstrated in the phase 1 studies (12). For all doses except 25 mg QD receptor saturation of >80% was observed throughout the dosing period. However, there was no correlation between viral load reduction and degree of receptor saturation. The most likely explanation for this is that very high levels of receptor saturation is required for antiviral efficacy and the inherent variability of the assay does not allow differentiation to that degree (11, 12).

**Figure 1:**
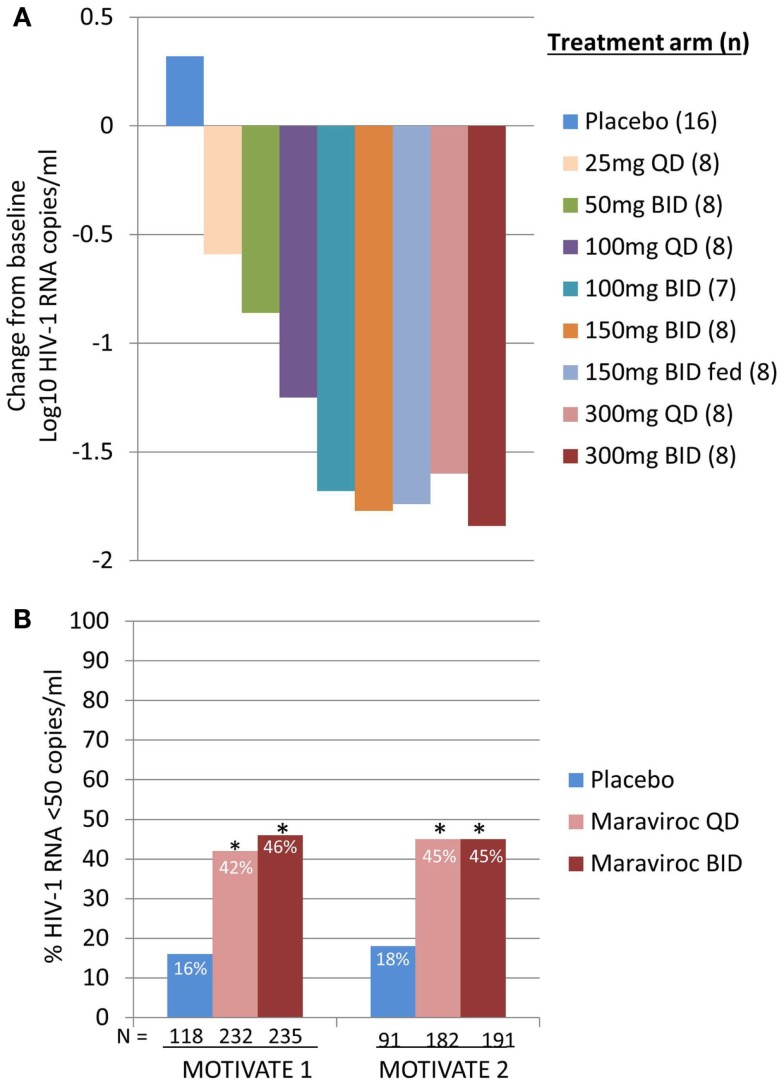
**Maraviroc proof of concept and phase 3 efficacy results**. **(A)** Mean maximum change from baseline in HIV-1 RNA in patients receiving MVC monotherapy. Based on phase 1 data and modeling and simulation, doses ranging from 25 mg QD to 300 mg BID (including 150 mg BID fed and fasted) were selected. HIV-1 RNA, safety, and MVC pharmacokinetics were evaluated (12). **(B)** MOTIVATE 1 and 2 – proportion of patients achieving HIV-1 RNA <50 copies/mL at week 48. HIV-1 infected patients with R5 HIV-1 and triple class experience and/or resistance were randomized to receive MVC QD or BID, or placebo, in combination with an optimized background antiretroviral regimen (OBT). *P* < 0.001 (13, 14).

The phase 2a data generated excitement throughout the company and we were keen to progress the clinical development program as quickly as possible as there was a high medical need for new ARVs to treat patients with no or limited treatment options. The extensive phase 1 program (including multiple drug-drug interaction studies) and wide dose range evaluated in the phase 2a proof of concept studies, together with modeling and simulation, gave us a very good understanding of the likely efficacious dose of MVC in combination with other ARVs. We were therefore able to move straight to phase 3 efficacy studies evaluating MVC at 300 mg (or equivalent, depending on co-administered drugs) QD and BID, without the need to do stand-alone phase 2b dose-ranging studies, thereby significantly shortening the development timeline. In late 2004, we initiated four large studies; MOTIVATE 1 and 2 in treatment-experienced patients with R5 HIV-1 (13, 14), MERIT (a phase 3 study with a phase 2b roll-in) in treatment-naïve patients with R5 HIV-1 (15), and study A4001029, a phase 2b safety study in treatment-experienced patients with non-CCR5 tropic virus (CXCR4-using or non-phenotypable virus) (16).

This was a massive undertaking, with 4794 patients screened at more than 200 sites in the USA, Canada, Europe, Australia, South Africa, Mexico, and Argentina. Two other small molecule CCR5 antagonists (aplaviroc and vicriviroc) were also being evaluated in phase 2b studies at this time (17, 18). In addition to the usual challenges of managing large clinical studies, we were thrown two curveballs, the first of these were the discontinuation of aplaviroc due to idiosyncratic hepatotoxicity. There was speculation that this could be a class effect of CCR5 antagonists as CCR5 knockout mice are more susceptible concanavalin-A mediated hepatoxicity (17). Additionally, a patient in the MERIT study developed severe hepatotoxicity. The data implied that it was likely related to isoniazid or cotrimoxazole, but a contributory role for MVC could not be excluded (15). An in-depth review of all data for evidence of hepatotoxicity for MVC and a high level of vigilance for any signals, did not find any evidence for a systematic increase in hepatic enzymes or other markers for hepatotoxicity. Shortly afterwards concerns were raised regarding a potential increased risk for certain malignancies, following the occurrence of lymphoma in four patients receiving vicriviroc in study ACTG5211 (18). Initially there were concerns that this could be a class-effect based on the immune-modulatory potential of CCR5 antagonists, but review of data from other vicriviroc studies, as well as the ongoing MVC studies did not support this theory (18).

Data from MOTIVATE 1 and 2 and A4001029 were available ahead of that of MERIT, as study duration is typically shorter for studies in treatment-experienced patients. It was with great excitement that we awaited the week 24 interim analyses for the MOTIVATE studies in October 2006 and we were elated to see that significantly more patients receiving MVC had an HIV-1 RNA of <50 copies/mL (the key marker for efficacy) compared to those receiving placebo OBT. This was confirmed by the week 48 data, demonstrating durability of response (Figure 1B) (13, 14). In contrast, patients with non-CCR5 tropic HIV-1 receiving MVC in A4001029 did not appear to gain significant virologic benefit compared to placebo (16). Analysis of safety data raised no significant concerns. Specifically, there was no evidence of an adverse effect on immune function, with no increase in episodes of infection or malignancies in MVC treated patients. Assessment of virus tropism at failure demonstrated that >50% of patients failing MVC therapy had CXCR4-using virus at failure, but there was no evidence of a deleterious effect on CD4 cell count numbers (14). Virologic assessment demonstrated that the CXCR4-using virus that emerged under MVC selective pressure was from a pre-existing minority population and did not arise *de novo* (19). Altogether, these results clearly demonstrated the benefit of MVC in the management of treatment-experienced patients with R5 HIV-1. A supreme effort by the team resulted in submission of dossiers for registration in both the USA and Europe only 2 months after the interim data became available. MVC (300 mg BID) received approval for use (in combination with other ARVs) in the USA in August 2007, only 6.5 years after it was nominated as a candidate for clinical development. One month later, it was also approved for use in this population in the EU.

The week 48 analysis of the MERIT study was disappointing, as MVC plus zidovudine/lamivudine (HIV-1 RNA <50 copies/mL, 65.3%) did not meet the pre-set criteria for non-inferiority (lower bound of the 1-sided 97.5 confidence interval below −10%) to efavirenz plus zidovudine/lamivudine (HIV-1 RNA <50 copies/mL, 69.3%) (15). However, patients for this study were screened for R5 virus using the original Trofile assay. This assay has been improved in the meantime to be more sensitive for the detection of minority populations of CXCR4-using virus. All screening samples for patients in MERIT were subsequently retested using the enhanced assay and a *post hoc* analysis performed including only patients who had R5 virus only by the more sensitive assay. In this analysis the response rates for MVC and efavirenz were 68.3 and 68.5%, respectively, with the lower bound of the 97.5% confidence interval above −10% (15). Based on this data, MVC was also approved for use in treatment-naïve patients in November 2009 by the United States Food and Drug Administration.

MVC has not only proved to be a valuable addition to the ever growing ARV drug armamentarium, but data from these studies have improved our understanding of HIV tropism and the relationship between tropism and disease progression. For me, personally this represented a period of great excitement and satisfaction, both as a physician and scientist.

## Author Contributions

ER drafted this manuscript based on her personal experience as a member of the MVC development team. All the data have been published in full elsewhere. All studies were conducted in compliance with the principles of the Declaration of Helsinki and with all International Conference on Harmonization Good Clinical Practice Guidelines and local regulatory and legal requirements. All studies were approved by independent ethics committees and all patients gave written informed consent.

## Conflict of Interest Statement

Elna Van Der Ryst was an employee of Pfizer Global Research and development at the time MVC was developed. She currently provides consulting services to Pfizer.
